# Extrathoracic investigation in adult patients with isolated pulmonary langerhans cell histiocytosis

**DOI:** 10.1186/s13023-016-0387-1

**Published:** 2016-02-02

**Authors:** Abdellatif Tazi, Constance de Margerie-Mellon, Laetitia Vercellino, Jean Marc Naccache, Stéphanie Fry, Stéphane Dominique, Stéphane Jouneau, Gwenaël Lorillon, Emmanuelle Bugnet, Raphael Chiron, Benoit Wallaert, Dominique Valeyre, Sylvie Chevret

**Affiliations:** Assistance Publique-Hôpitaux de Paris, Hôpital Saint-Louis, Centre National de Référence de l’histiocytose Langerhansienne, Service de Pneumologie, 1 Avenue Claude Vellefaux, 75475 cedex 10 Paris, France; Université Paris Diderot, Sorbonne Paris Cité, U1153 CRESS, Biostatistics and Clinical Epidemiology research team, Paris, France; Assistance Publique-Hôpitaux de Paris, Service de Radiologie, Hôpital Saint-Louis, Paris, France; Assistance Publique-Hôpitaux de Paris, Service de Médecine Nucléaire, Hôpital Saint-Louis, Paris, France; Assistance Publique-Hôpitaux de Paris, Service de Pneumologie, Hôpital Avicenne, Bobigny, France; Service de Pneumologie et Immuno-allergologie, Centre de compétence des maladies pulmonaires rares, Hôpital Calmette, Lille, France; Département de Pneumologie, Hôpital Charles Nicolle, Rouen, France; IRSET UMR 1085, Université de Rennes 1; Service de Pneumologie, Hôpital Pontchaillou, Rennes, France; Département de Pneumologie, Hôpital Arnaud de Villeneuve, Montpellier, France; Université Lille 2, Lille, France; Université Paris 13, Sorbonne Paris Cité; Assistance Publique-Hôpitaux de Paris, Service de Pneumologie, Hôpital Avicenne, Bobigny, France; Assistance Publique-Hôpitaux de Paris; Hôpital Saint-Louis, Service de Biostatistique et Information Médicale, Paris, France

**Keywords:** Langerhans cell histiocytosis, Bone x-ray, Bone scan, Blood cell count, Liver biology, Management

## Abstract

**Background:**

An important objective on diagnosis of patients with Langerhans cell histiocytosis (LCH) is to determine the extent of disease. However, whether systematic extrathoracic investigation is needed in adult patients with clinically isolated pulmonary LCH (PLCH) has not been evaluated.

**Methods:**

In this prospective, multicentre study, 54 consecutive patients with newly diagnosed clinically isolated PLCH were systematically evaluated at inclusion by bone imaging and blood laboratory testing to search for subclinical extrapulmonary LCH involvement. The patients were followed over a 2-year period. At each visit, they were asked about the presence of extrapulmonary manifestations of LCH.

**Results:**

In the absence of bone symptoms, the skeletal X-ray survey results were normal for all but two patients who had a localised bone lesion consistent with possible LCH involvement, that remained unchanged over 2 years of follow-up. Whole-body bone scintigraphy did not add information to the plain radiography findings for the detection of asymptomatic bone involvement in isolated PLCH. Conversely, it showed nonspecific focal bone uptake in 18 % of the patients, mainly corresponding to post-traumatic or degenerative abnormalities unrelated to LCH. Mild leucocytosis due to neutrophilia was observed in 22 % of the patients and was not related to their smoking habits. Three patients had mild isolated lymphocytosis without haematological disease, whereas two patients had mild lymphopaenia. A mild inflammatory biological syndrome was observed in a minority of patients without infection or constitutional symptoms and was not associated with progressive disease. A substantial proportion (24.5 %) of the patients had abnormal biological liver test results, including elevated liver enzymes and/or cholestasis, which were not linked to LCH involvement in this cohort.

**Conclusions:**

Obtaining a thorough history and performing comprehensive physical examination are essential for staging patients diagnosed with PLCH. In the absence of symptoms or signs suggestive of extrapulmonary LCH involvement, the systematic performing of recommended bone imaging does not appear informative. Although the observed blood laboratory abnormalities were not specifically related to LCH, performing these tests in the diagnostic workup for PLCH is useful because some of these alterations may impact patient management.

**Trial registration:**

ClinicalTrials.gov: No. NCT01225601; URL: www.clinicaltrials.gov

## Background

Adult pulmonary Langerhans cell histiocytosis (LCH) is a rare disorder of unknown aetiology that occurs predominantly in young smokers, with an incidence peak at 20–40 years of age [1, 2]. Although pulmonary involvement in LCH may be part of a multisystem disease [3–5], pulmonary LCH (PLCH) occurs frequently as a single-system disease in adults [1, 2].

An important objective upon LCH diagnosis is to determine the extent of disease [6]. Therefore, obtaining a thorough history and performing a comprehensive clinical evaluation, several laboratory tests, and bone imaging are recommended for all adult LCH patients, regardless of the primary manifestation of the disease [6]. However, whether a similar approach is needed for adult patients with clinically isolated PLCH has not yet been evaluated. To address this issue, we examined data from our prospective multicentre study that evaluated the natural history of recently diagnosed PLCH [7], in which the patients systematically underwent bone imaging and blood testing at the time of inclusion.

## Methods

### Study design

This is an ancillary study derived from a recently reported prospective, multicentre study conducted by the French National Reference Centre for Langerhans Cell Histiocytosis, in which the natural history of adult PLCH patients was evaluated [7]. The study protocol was approved by the appropriate ethics committee in February 2006 (N°2006/03, CCPPRB IDF4, Paris). Consecutive patients who were 18 years of age or older and were referred for PLCH to the participating centres were considered eligible, provided they had not received treatment for their disease. The main exclusion criterion was the presence of extrapulmonary involvement of LCH, except for localised bone lesions. All patients provided written informed consent.

At the time of inclusion in the study, a comprehensive clinical evaluation, including a thorough history and a search for the presence of respiratory and constitutional symptoms (e.g., fever, night sweats, and body weight changes) and extrapulmonary manifestations of LCH were performed. In addition, bone imaging and blood laboratory testing were systematically conducted. Because head MRI is not systematically recommended [6], it was not included in the imaging workup.

### Study subjects

Among the 58 patients included in our initial study [7], four were secondarily excluded because of symptomatic LCH bone involvement detected upon PLCH diagnosis (*n* = 3) or at the time of inclusion (*n* = 1). They complained of bone pain and had typical LCH osteolytic lesions. In two of these patients, bone involvement was the first sign of LCH and was histologically confirmed. The characteristics of the remaining 54 patients with clinically isolated PLCH at the time of inclusion are shown in Table 1. No patient had symptoms or signs suggestive of extrapulmonary LCH. The diagnosis of PLCH either was histologically confirmed by a surgical lung biopsy (*n* = 19) or was based on the combination of an appropriate clinical setting, a typical lung high-resolution computed tomography (HRCT), a marked predominance of alveolar macrophages in bronchoalveolar lavage, and exclusion of alternative diagnoses (*n* = 35) [7].

**Table 1:** Baseline characteristics of the patients at inclusion in the study

Characteristic	*N* = 54
Age, yrs, median, [IQR]	35.5 [27–42]
Female sex, n (%)	30 (56 %)
Smoking history, pack-years Smoker, n (%) Ex-smoker	16.5 [9–25] 38 (70 %) 16 (30 %)
Clinical features, n (%)	
Asymptomatic	19 (35 %)
Respiratory symptoms (cough, dyspnoea)	31 (57 %)
History of pneumothorax	11 (20 %)
Constitutional symptoms*	6 (11 %)

*Abbreviation definitions*: *IQR* interquartile range

*Constitutional symptoms were associated with respiratory symptoms in four patients

### Bone imaging

Skeletal X-ray survey was performed, consisting of anteroposterior and lateral views of the skull and spine, as well as anteroposterior views of the ribs, pelvis, and upper and lower limbs. A dental panoramic radiograph was also obtained.

All patients had a chest CT at the time of inclusion as part of pulmonary evaluation [7], and eventual LCH involvement of the thoracic bones was systematically assessed by analysing CT images with bone window settings (width: 2000, level: 300).

Whole-body bone scintigraphy was also systematically performed at inclusion. The patients were injected intravenously with technetium (Tc)-99 m-radiolabelled bisphosphonates. At least 2 h after tracer injection, total body imaging (planar anterior and posterior views) was performed with a dedicated dual-head gamma camera. Complementary static images were captured only if deemed necessary.

### Blood laboratory tests

At inclusion, a complete blood count (haemoglobin, white blood cell and differential counts and a platelet count) and blood chemistry analysis (total protein, electrolyte, creatinine, bilirubin, alanine aminotransferase (ALT), aspartic aminotransferase (AST), alkaline phosphatase (AP), gammaglutamyl transpeptidase (γGT), C-reactive protein (CRP), and fibrinogen levels) were performed. In our experience, all adult patients with LCH pituitary involvement and diabetes insipidus complain of polyuria/polydipsia. Therefore, morning urinary osmolarity was not included in the biological workup.

### Data collection

The patients were managed as outpatients at each study centre. Study visits occurred at baseline and at 3, 6, 12, 18, and 24 months. A standardised case report form was completed at each investigation centre. The data were monitored by independent clinical research assistants. At each visit, the presence of respiratory, constitutional and extrapulmonary manifestations of LCH was recorded. Bone radiographs and ^99m^-Tc bone scans were interpreted by expert radiologists and nuclear physicians, respectively, at each participating centre. Lung CT scans were centrally analysed by a radiologist (C de M). The blood laboratory test results were interpreted according to the normal reference values. In cases of abnormal findings, additional investigations were carried out as needed to establish a cause of the abnormality and to manage the patient.

### Statistical analysis

Descriptive statistics are presented as median (interquartile range [IQR]) values. Comparisons between the groups at inclusion, as defined by the blood leucocyte count or biological inflammation, were conducted using Fisher’s exact test or the nonparametric Wilcoxon test for qualitative and quantitative variables, respectively.

All statistical analyses were performed using SAS 9.3 (SAS Inc., Cary, NC, USA) and R 3.0.2 (http://www.R-project.org/). Two-sided P-values of less than 0.05 were considered statistically significant.

## Results

### Bone imaging findings

Fifty-one (94.4 %) patients received skeletal X-ray survey at inclusion. One patient had a lytic lesion of the left femoral diaphysis, confirmed by bone MRI, which was consistent with possible LCH involvement. During the 24 months of follow-up in the study, this patient did not present bone symptoms, the femoral lesion remained stable, and no other bone localisation appeared. Another patient had fracture sequelae of the 8th right rib on plain radiography that was also visible on chest CT. This lesion remained unchanged at 2 years of follow-up. Because these two patients had no symptoms, the eventual LCH bone involvement was not confirmed by a bone biopsy. The 49 remaining patients had normal skeletal X-ray survey findings. With the exception of the patient with fracture sequelae of the 8th right rib, no patient had a thoracic bone lesion on chest CT.

Of the 47 (87 %) patients who received dental panoramic radiography, 44 had normal findings. Three patients exhibited extensive loss of the teeth, without mandible/maxillary alveolar or basal bone lytic lesions. These patients were referred to the maxillofacial surgery clinic and none complained of previous or current dental or oral mucosa symptoms suggestive of LCH. It was concluded that the previous extensive tooth extractions were related to poor oral hygiene and periodontitis favoured by their heavy smoking habits.

All 54 patients underwent whole-body bone scintigraphy at inclusion. The bone scan was normal in 43 (80 %) patients, including the patient with the lytic lesion of the left femoral diaphysis. The patient with fracture sequelae of the 8th right rib showed mild focal ^99m^-Tc uptake. For the 10 (18 %) remaining patients, bone scan showed mild focal ^99m^-Tc uptake at different skeletal sites, whereas skeletal X-rays showed no histiocytosis-related lesions in the corresponding areas (Table 2).

**Table 2:** ^99m^-Tc bone scintigraphy for the 10 PLCH patients with normal skeletal X-ray survey at inclusion

Patient	^99m^-Tc bone scintigraphy findings
1	Focal uptake of proximal right humerus (post-traumatic)
2	Uptake of lateral part of L4-L5
3	Mild uptake of 4^th^ left rib
4	Moderate uptake of 9^th^ right rib
5	Mild uptake of proximal right tibia
6	Mild uptake of lateral part of left C7
7	Uptake of right C7
8	Uptake of right lateral part of L1
9	Uptake of right lateral part of D11
10	Focal uptake of left patella and upper part of right orbit

*Abbreviation definitions*: *L* lumbar vertebra, *C* cervical vertebra, *D* dorsal vertebra

### Blood laboratory test findings

All patients had normal blood total protein, electrolyte and creatinine levels. Twelve (22 %) patients had an increased white blood cell count (median = 12.75 G/L, IQR 12-13.3 G/L; normal range 4–10 G/L). Among these patients, 11 had neutrophilia (median = 8.9 G/L, IQR 8.5-9.5 G/L; normal range 1.7–8.0 G/L), and two had an associated mild increase in the lymphocyte count (5.9 G/L, and 4.8 G/L, respectively; normal range 1.5–4.0 G/L). Another patient had mild isolated lymphocytosis (5.3 G/L). No association was observed with smoking status (current vs. ex-smoker) at inclusion (*p* = 0.73), daily cigarette consumption (*p* = 0.98) or cumulative tobacco smoking, expressed as pack-year (*p* = 0.37). Additionally, two patients presented mild lymphopaenia (1.3 and 1.1 G/L, respectively) with a normal total blood count. These two patients had no other cause of lymphopaenia.

A mild inflammatory biological syndrome was observed in 5 patients (CRP = 29 to 49 mg/L, *n* = 4; normal value < 10 mg/L; fibrinogen = 4.6 to 7.3 g/L, *n* = 3; normal range 2–4 g/L). None of these patients had constitutional symptoms or progressive disease. No association was observed with smoking status (*p* = 0.31).

Thirteen of the 53 patients (24.5 %) had abnormal biological liver test results, including elevated liver enzymes and/or cholestasis. All these patients were referred to the hepatology department at their investigating centre. Additional tests, including hepatitis virus serology and auto-immune antibody testing, hepatic imaging (ultrasound and MR cholangiography), and, in one case, liver biopsy, were performed. The biological liver abnormalities were not related to LCH involvement in any of the cases. The diagnoses retained for the biological liver abnormalities in these patients are shown in Table 3.

**Table 3:** Retained diagnoses for the 13 PLCH patients with abnormal liver biological test results at inclusion

Patient	AST	ALT	AP	γGT	Diagnosis
1	N	N	N	2.3 N	Alcohol consumption
2	N	N	2 N	4 N	Alcohol consumption (negative results for hepatitis virus serology, auto-antibodies and MR cholangiography)
3	N	N	1.3 N	1.8 N	Drug induced
4	N	N	N	2.5 N	Liver steatosis
5	1.3 N	1.2 N	N	4.9 N	Alcohol consumption
6	N	N	N	6 N	Alcohol consumption
7	1.9 N	2 N	N	6 N	Alcohol consumption
8	N	1.2 N	N	2 N	Alcohol consumption
9	N	1.5 N	N	2 N	Liver steatosis
10	N	2 N	1.3 N	2 N	No diagnosis, lost to follow-up
11	N	N	N	2 N	Liver steatosis
12	N	N	N	3.6 N	Alcohol acute pancreatitis
13	N	N	N	1.7 N	Alcohol consumption

*Abbreviation definitions: AST* aspartic aminotransferase, *ALT* alanine aminotransferase, *AP* alkaline phosphatase, *γGT* gammaglutamyl transpeptidase, *N* normal value, *MR* magnetic resonance

## Discussion

This is the first study to evaluate the utility of systematic extrathoracic examination in adult patients with clinically isolated PLCH. We report the following findings: 1) in the absence of bone symptoms, skeletal X-ray survey was virtually consistently normal; 2) bone scan did not add information to plain radiography and showed nonspecific focal ^99m^-Tc uptake corresponding to post-traumatic or degenerative lesions in a significant minority of the patients; and 3) routine biological blood tests showed various abnormalities that were not specifically related to LCH.

Bone involvement is known to occur in adult PLCH [1, 2]. Several retrospective studies have demonstrated that bone involvement occurs in approximately 10 % of adult PLCH patients [8–12]; however, these studies did not specify whether the patients had bone symptoms. In a former large retrospective series, Friedman et al. “incidentally” detected bone involvement in only 4 out of 100 PLCH patients [13]. Among a multicentre prospective cohort of 77 PLCH patients, Schönfeld et al. identified eight (10 %) with clinical signs of bone involvement at the time of diagnosis [14]. In a recent single-centre series of 40 PLCH adult patients, two (5 %) had symptomatic bone involvement at diagnosis, and seven (18 %) had radiographic LCH bone involvement [15]. However, it is unclear whether all of the patients underwent skeletal radiography at diagnosis and if the bone radiographs were obtained because of the occurrence of bone symptoms during follow-up.

Because LCH may involve the jaw [5, 16], dental panoramic radiography was included as part of bone imaging at inclusion in our study, showing normal results for all of the patients except for three who presented extensive tooth loss. None of these patients exhibited mandible/maxillary lytic lesions of the alveolar or basal bones. These patients did not complain of previous dental or oral signs suggestive of LCH. They had previous extensive dental extractions related to poor oral hygiene and periodontitis favoured by their heavy smoking habits.

The contribution of bone scanning to the identification of LCH bone lesions has been controversial for a long time, and appears to be less sensitive than plan radiography [17–19]. Our results clearly demonstrate that bone scanning does not provide any added contribution to skeletal radiography for detecting asymptomatic bone involvement in PLCH. Whole-body bone scintigraphy did not identify the femur lytic lesion observed on skeletal X-ray. Conversely, bone scan showed nonspecific focal ^99m^-Tc bone uptake in a substantial proportion of the patients, mainly corresponding with post-traumatic or degenerative abnormalities unrelated to LCH. None of these patients developed secondary LCH bone involvement.

^18-FDG^PET-CT was shown to be more sensitive that skeletal X-ray survey to detect bone LCH, particularly some localisations such as rib and pelvic lesions [20]. Furthermore, FDG uptake was correlated with the activity of bone disease and was useful for following the disease course and evaluating response to treatment [20]. However, whether ^18-FDG^PET-CT should be systematically performed as part of diagnostic workup for patients with clinically isolated PLCH (i.e. with no bone symptoms) is questionable and remains to be evaluated. The increased radiation exposure in these young patients is also a concern.

Whereas routine blood electrolyte and renal function testing showed consistently normal results in our cohort, a slightly increased blood leucocyte count was observed in 22 % of the patients. This leucocytosis was due to neutrophilia in all but one of the patients. Although smoking is known to induce leucocytosis [21], we did not find any statistical association between the patients’ blood leucocytosis and their smoking habits. Three patients (5 %) had mild lymphocytosis without haematological disease. Conversely, two patients had mild lymphopaenia, which is unusual in LCH patients in the absence of immunosuppressive therapy. No other causes of lymphopenia, such as HIV infection, were found.

A mild biological inflammatory syndrome may have been present in a small proportion of the patients with clinically isolated PLCH, in the absence of any constitutional symptoms or infection. This inflammatory syndrome was not associated with progression of the disease.

The liver biological blood tests revealed the most salient blood abnormalities in this study. Different patterns of biological liver abnormalities were observed, i.e., elevated liver enzymes and/or cholestasis. In our cohort, these abnormalities were not related to LCH liver involvement. The main aetiology of the biological liver abnormalities was excessive alcohol consumption. This possibly reflects the consumption level of the general French population or a particular addictive tendency of PLCH patients towards alcohol, in the same manner as that to tobacco [1, 2].

Although the laboratory blood abnormalities observed were not specifically related to LCH, in our opinion, these tests merit systematic administration at the time of PLCH diagnosis for several reasons. First, haematological disorders (leukaemia or lymphoma) may be associated with LCH, including adult PLCH [11, 22, 23], and they may thus be screened using these blood tests. Second, although it was not the case in our cohort, liver LCH involvement in adult PLCH patients has been reported [24–26]. Finally, the presence of lymphopaenia and biological liver alterations is important to consider in patients who will eventually need treatment for their disease. In particular, cladribine, a promising treatment for progressive PLCH [27, 28], is known to induce deep and prolonged lymphopaenia and hepatic side effects [29].

## Conclusions

Our results highlight the importance of obtaining a thorough history and performing comprehensive physical examination to search for extrapulmonary involvement in adult PLCH patients. In the absence of symptoms or signs suggestive of extrapulmonary LCH involvement, the systematic performing of recommended bone imaging does not appear informative. The prospective and multicentre design of our study enhances the external validity of this finding. Conversely, blood cell count and liver biological assessments should be performed in diagnostic workup because they can reveal abnormalities that have potential impacts on patient management. Additional studies are needed to determine if these biological tests should be systematically repeated during the follow-up of patients with normal initial assessment.

## Abbreviations

ALT: alanine aminotransferase
AP: alkaline phosphatase
AST: aspartic aminotransferase
CRP: C-reactive protein
CT: computed tomography
IQR: interquartile range
LCH: langerhans cell histiocytosis
MRI: magnetic resonance imaging
SD: standard deviation
γGT: gammaglutamyl transpeptidase
